# Symptoms of anxiety disorders in Iranian adolescents with hearing loss during the COVID-19 pandemic

**DOI:** 10.1186/s12888-021-03118-0

**Published:** 2021-02-22

**Authors:** Saeed Ariapooran, Mehdi Khezeli

**Affiliations:** 1 Department of Psychology, Malayer University, Malayer, Iran; 2 Social Development and Health Promotion Research Center, Health Institute, Kermanshah University of Medical Sciences, Kermanshah, Iran

**Keywords:** Adolescents, Anxiety, COVID-19 pandemic, Deaf, Hearing loss, SCARED

## Abstract

**Background:**

Anxiety symptoms have been reported in many populations during the COVID-19 pandemic, but not in adolescents with a hearing loss. This study aimed to investigate the presence of symptoms of anxiety disorders (ADs) in adolescents with hearing loss (HL) during the COVID-19 pandemic, 2020.

**Methods:**

In this cross sectional study, 56 adolescents with HL (aged 12 to 18 years) including 23 deaf, and 33 hard of hearing (HH) were selected from four counties located in western Iran using a census method. Adolescents with HL filled out the self-report of the Screen for Child Anxiety Related Emotional Disorders (SCARED).

**Results:**

The results showed that the presence of symptoms of ADs in adolescents with HL was 37.5%, and higher in deaf than in HH adolescents (60.9% in deaf vs. 21.2% in HH, *p* = 0.003). Among the subscales, only the Social Anxiety Disorder (39.1% in deaf vs. 90.1 in HH, *p* = 0.009) and the School Avoidance (52.2% in deaf vs. 24.2% in HH, *p* = 0.031) significantly differed. The mean score of Panic Disorder, Social Anxiety Disorder, and Anxiety Disorders in the deaf adolescents were higher than in HH ones.

**Conclusions:**

Our study showed the presence of significant symptoms of ADs in a sample of Iranian adolescents with HL, especially in deaf adolescents, during the COVID-19 pandemic.

## Background

COVID-19 is an acute respiratory illness caused by a new coronavirus (SARS-CoV-2), which quickly spread around the world [1]. According to the World Health Organization, Iran is the leading country in the number of cases and deaths due to Quid 19 in the Eastern Mediterranean region [2].

Fear and anxiety are adaptive reactions to unknown conditions such COVID-19 pandemic [3]. This is probably one of the reasons that psychological disorders were commonly reported during the COVID-19 pandemic [4]. Anxiety symptoms have been reported in many populations around the world during the COVID-19 pandemic [5–7]. In Iran, high levels of anxiety symptoms have been also reported in different populations [8, 9]. A comparative study found that COVID-19 pandemic significantly increased anxiety symptoms in the general United States population compared to pre-pandemic with a relative risk of 3.77 [10]. In a review study, Fardin (2020) showed that the COVID-19 pandemic is associated with negative psychological effects such as increased anxiety [11].

Worldwide 466 million people (6.6%) live with HL, of which 34 million are children. HL may be mild, moderate, severe, or profound; it can affect one or both ears, causing difficulty in hearing a conversation or in hearing intense sounds [12]. Hard of hearing (HH) refers to people with hearing problems from mild to severe. Hard of hearing people usually communicate through spoken language and can use hearing aids such as cochlear implants. Deaf people have profound HL and hear very little or do not hear at all and often use sign language to communicate [12].

Hearing loss can have a profound effect on people’s mental health and alter some psychological and social disorders [13, 14]. Some psychological conditions such as depression, anxiety, and personality disorders can even lead to serious outcomes such as self-harm and suicide attempts [15, 16], especially in people with HL [17, 18]. Some studies have suggested that COVID-19 can affect the mental health of individuals and in interaction with socio-economic factors can lead to cases of suicide attempts in individuals [19–22]. Anxiety is one of the most common disorders associated with hearing loss. In a systematic review, Shoham et al. [23] showed that people with HL reported high levels of anxiety disorders (ADs). Studies have also showed that symptoms of anxiety in people with HL were higher than in their hearing peers. For example, Hallam et al. reported that 30.4% of people with hearing loss were anxious compared to 10.4% of the general UK population [24]. Ariapooran indicated that 15.15% of deaf children, and 8.3% of HH children had symptoms of ADs. He also showed that the symptoms of ADs, especially panic disorder (PD), GAD, and SAD, were higher in deaf than HH children, while SoAD was higher in HH [25]. Kvam and her colleagues reported that whereas 1% of the respondents in general population were anxious, the percentage among the deaf respondents was 10% [26]. Defect in language skills due to the hearing loss can affect the communication, and consequently can have a devastating effect on emotional and psychosocial functioning [27].

The rate of ADs in adolescents with HL during the COVID-19 pandemic has not been studied. Since the information and resources related to the COVID-19 disease are not readily available to the people with HL, especially deaf people [28], it can be argued that these individuals may experience psychological problems, including symptoms of ADs, more than others during the outbreak of the COVID-19. Accordingly, the present study aimed to investigate the symptoms of ADs in adolescents with HL during the outbreak of the COVID-19 in Iran.

## Methods

### Sample and procedure

This was a cross-sectional, comparative study because examined the symptoms of ADs in deaf and HH adolescents during the COVID-19 pandemic. The sample was comprised of all adolescents aged 12–18 that had HL including deaf and HH (with HL ranging from mild to severe) in four counties of Iran: Borujerd (in Lorestan province, 12 adolescents; 7 HH and 5 deaf); Malayer (in Hamadan province, 13 adolescents; 8 HH and 5 deaf); Nahavand (in Hamadan province, 15 adolescents; 9 HH and 6 deaf); and Tuyserkan (in Hamadan province, 16 adolescents; 9 HH and 7 deaf). All of these HL adolescents attended exceptional schools. Sampling was done by a census method due to the limited number of HL adolescents. A total of 56 students (33 HH and 23 deaf students) participated in the study. We called parents by a phone and went to their home after getting permission. Afterward, the explanations and study objectives were provided, the inform consent was obtained, and the questionnaires were completed (see below). Adolescents filled out the paper questionnaires in their home within 35 to 45 min. The assessments were organized during April and January 2020.

### Questionnaire of screen for child anxiety related emotional disorders (SCARED)

The SCARED [29] contains 41 items, which examine various anxiety related emotional disorders and all are scored on a 3-point Likert scale (very true or often true = 2 to hardly true or not true = 0) that. A total score is a sum of all answered items and can range from 0 to 82. The questionnaire has five subscales: Panic Disorder (PD; 13 items), Generalized Anxiety Disorder (GAD; 9 items), Separation Anxiety Disorder (SAD; 8 items), Social Anxiety Disorder (SoAD; 7 items), and School Avoidance (SA; 4 items). The cut-off point for the overall ADs is a score ≥ 25 [21]. Scores ≥7 in the PD subscale, ≥ 9 in the GAD subscale, ≥ 5 in the SAD subscale, ≥ 8 in SoAD subscale, and ≥ 3 in the SA subscale are considered as a cut-off point for each of these symptoms. The validity assessed showed a sensitivity of .71 for the questionnaire and its internal consistency was reported to be between .78 and .87, and test-retest between .70 and .90 [29]. In Iran, the test-retest reliability of the PD, GAD, SAD, SoAD and SA were .83, .78, .81, .78 and .80 respectively and for the whole score .83 [26]. Jalali et al. (2018) found Cronbach’s alpha coefficients of .78, .71, .73, .76, and .61 for PD, GAD, SAD, SoAD, and SA, respectively and .98 for the whole scale [30]. In the present study the Cronbach’s alpha coefficients of the entire questionnaire was .81, and for GAD, SAD, PD, SA, and SoAD was .77, .47, .36, .40, and .74, respectively.

### Ethical aspect of the study

Confidentiality of information was guaranteed to all participants, and written inform consent form was obtained from students and their parent. This study received ethics approval from the Research Ethics Committee of Malayer University (IR.MALAYERU.REC.1399.007). We confirm that all methods related to the human participants were performed in accordance with the Declaration of Helsinki and approved by Research Ethics Committee of Malayer University.

### Data analysis

Based on the cut-off point for the ADs, descriptive statistics (frequencies, percent, mean, and standard deviation) were provided. We used independent t-test for comparing the presence of ADs in deaf and HH adolescents. The IBM SPSS Statistics version 19 was used to data analysis.

## Results

### Description of the sample

The mean (SD) age of the participants was 15.52 (1.91). Of the sample, 21 students (37.5%) were females (14 HH and 7 deaf) and 35 (62.5%) were males (19 HH and 16 deaf). Among the deaf adolescents, 18 (78.3%) were pre-lingually deaf and 5 (21.7%) were post-lingually deaf. Of the HH adolescents, 23 (69.7%) used hearing aids.

### Symptoms of ADs

The mean and standard deviation of the examined variables are reported in Table 1. The results showed that the mean score of ADs (*t* = 4.35, *P* < .001), PD (*t* = 2.62, *P* = .011), and SoAD (*t* = 3.67, *P* < .001) were higher in deaf adolescents than in HH ones. There was no significant difference in the mean score of other variables between the two groups.

**Table 1:** Mean (SD) of the variables in deaf and HH sample of study

Variables	Groups	*N*	Mean	SD	t	*p*	Effect size
**PD**	deaf	23	5.96	2.10	2.62	.011	.11 3
	HH	33	4.51	1.97			
**GAD**	deaf	23	6.48	3.20	1.62	.109	.047
	HH	33	5.15	2.85			
**SAD**	deaf	23	4.78	1.44	1.61	.112	.046
	HH	33	3.97	2.08			
**SoAD**	deaf	23	6.70	2.38	3.67	001	.200
	HH	33	4.48	2.09			
**SA**	deaf	23	2.70	1.61	1.37	.174	.034
	HH	33	2.18	1.18			
**ADs**	deaf	23	26.61	5.25	4.35	.000	.260
	HH	33	20.30	5.39			

*PD* Disorder, *GAD* Generalized Anxiety Disorder, *SAD* Separation Anxiety Disorder, *SoAD* Social Anxiety Disorder, *SA* School Avoidance, *ADs* Anxiety Disorders: Panic, *HH* Hard of Hearing

Table 2 shows the presence of symptoms of ADs and its subscales in adolescents with HL during the COVID-19 pandemic based on the specific cut-off values. The results showed that 37.5% of HL adolescents had symptoms of ADs (60.9% in deaf vs. 21.2% in HH, *p*-value = 0.003). Also, symptoms of ADs sub-scales were as follow: 23.2% PD, 32.1% GAD, 39.3% SAD, 21.4% SoAD, and 35.7% SA. Among the subscales only SoAD (39.1% in deaf vs. 90.1% in HH, *P*-value = 0.009) and SA (52.2% in deaf vs. 24.2% in HH, *p*-value = 0.03) significantly were higher in the deaf adolescent than HH peers.

**Table 2:** Percentages of adolescents with ADs symptoms

Variables	Deaf (*n* = 23)	HH (*n* = 33)	All (*n* = 56)	χ^2^ test (*p* value)
**PD**	8 (34.78%)	5 (15.15%)	13 (23.21%)	2.93 (0.083)
**GAD**	10 (43.48%)	8 (24.24%)	18 (32.14%)	2.299 (0.111)
**SAD**	11 (47.83%)	11 (33.33)	22 (39.29%)	1.194 (0.208)
**SoAD**	9 (39.13%)	3 (9.09%)	12 (21.43%)	7.264 (0.009)
**SA**	12 (52.17%)	8 (24.24%)	20 (35.71%)	4.606 (0.03)
**ADs**	14 (60.87%)	7 (21.21%)	21 (37.5%)	9.095 (0.003)

*PD* Disorder, *GAD* Generalized Anxiety Disorder, *SAD* Separation Anxiety Disorder, *SoAD* Social Anxiety Disorder, *SA* School Avoidance, *ADs* Anxiety Disorders: Panic, *HH* Hard of Hearing

## Discussion

According to our results, more than a third of HL adolescents had symptoms of ADs. Many studies showed different anxiety symptoms in various group of population during the COVID-19 pandemic [5–7]. Other studies on children and adolescent have been done before the COVID-19 pandemic. A study in western Iran found that 10.7% of children and adolescents had symptoms of anxiety (according to the SCARED questionnaire) [31]. Hajiamini et al. also reported an overall 21% prevalence of an anxiety condition in Iranian children [32]. Safavi et al. have found that total anxiety disorders were the most prevalent group of psychiatric disorders (21.9%) in a sample of children and adolescents aged 6–18 years from Chaharmahal and Bakhtiari province, Iran [33]. Therefore, the high rate of ADs symptoms in sample of this study requires special attention especially considering its synchronicity with the COVID-19 pandemic. However, one of the main problems in examining the association of the COVID-19 pandemic with anxiety in people with hearing loss is that there are no comparative studies before and after the pandemic in this particular group. Uncertainty and novelty of COVID-19 pandemic, immediate transmission, increasing mortality rate, and the high rate of infection, may be raised concerns and anxiety about the future [34].

In our results, the percent of deaf adolescents with symptoms of ADs, SoAD, and SA were higher than the percentage of the HH ones. Studies prior to the COVID-19 pandemic also reported symptoms of social anxiety disorder in the deaf and HH adolescents. Azab et al. (2015) in a study before the COVID-19 pandemic found that SoAD and GAD subtypes of SCARED in children with mild hearing impairment were significantly lower than moderate and severe hearing impairment [35]. Dehghan and colleagues also referred to the high SA in deaf adolescent in Kermanshah, western Iran [36]. A systematic review study also found that deaf and hard of hearing people had higher symptoms of anxiety than hearing people [23]. However, the results of present study can be consistent with the theory of information deprivation trauma proposed by Schild and Dalenberg [37], according to which traumatization is a result of inadequate information in deaf people. It should also be noted that HL in deaf peoples may lead to the misinterpretation of verbal information about COVID-19 pandemic because it’s related information and resources are not readily available to the deaf people [28]. Maybe that’s why the WHO was asked to provide an international signing agreement related to the COVID-19 pandemic for deaf and hard of hearing people [38].

The results of the present study also showed that the mean score of PD, SoAD, and ADs in deaf were significantly higher than the HH adolescents. Also, after the overall score of ADs, which had the greatest size effect in distinguishing between deaf and HH groups, SoAD and PD among the dimensions of the questionnaire had the greatest effect on the distinction between the two groups, respectively. Theunissen et al. reported that anxiety in children with cochlear implants and normally hearing children were similar, while children with conventional hearing aids had higher social anxiety [39]. This is probably due to the fact that the severity of hearing loss is related to anxiety (especially social anxiety disorder) in children and adolescents. We also found that deaf adolescents had higher mean scores on the school avoidance subtype. Previous studies have shown that students with hearing impairments experience more emotional disorders than other students and have more school phobia [36, 40]. This study was conducted at a time when the presence of students in Iranian schools was not banned due to the COVID-19 pandemic, and perhaps one of the reasons for this result is the fear of deaf students attending schools due to the unknown nature of the disease, fear of infection, problems with wearing masks and gloves, lack of training materials related to COVID-19 disease for the deaf. Although this subtype of the questionnaire is not directly a symptom of anxiety disorder according to the DSM-IV-TR, it can be considered as a source of anxiety in adolescents with hearing impairment.

There are a number of limitations. One limitation of our study was the lack of a comparison group without HL. In our study, some of the symptoms rates were high and the mean of our variables was close to the cut-off point of the scales, so using a comparison group among adolescents without HL is suggested in future researches. Another limitation of the study was use of self-reports for assessing symptoms; therefore, we suggested the diagnostic interviews in future researches. It was also a cross-sectional observational study, which only allows correlation and not causal associations. We also had a small sample size from a selected area, which limit the generalizability to other region. In this study, only anxiety disorders were assessed and other psychopathologies and issues such as family and teacher interaction with students, stress, getting COVID 19 in family members and friends, post-traumatic stress disorder, and students’ risk perception were not examined.

## Conclusions

Our study showed the considerable of symptom of ADs and its sub-scales in a sample of Iranian adolescents with HL (especially deaf adolescents) during the COVID-19 pandemic. Therefore, the high rate of ADs symptoms in sample of this study requires special attention especially considering its synchronicity with the COVID-19 pandemic. Clinical psychologists, psychotherapists, and psychiatrists should pay more attention to ADs symptoms in adolescents with HL during the COVID-19 pandemic.

## Data Availability

The data sets used and analyzed in this study are available from the corresponding author on reasonable request.

## Abbreviations

ADs: Anxiety Disorders
GAD: Generalized Anxiety Disorder
HH: Hard of Hearing
HL: Hearing Loss
PD: Panic Disorder
SA: School Avoidance
SAD: Separation Anxiety Disorder SCARD
SCARD: Screen for Child Anxiety Related Emotional Disorders
SoAD: Social Anxiety Disorder
