# Correction to: Symptoms of anxiety disorders in Iranian adolescents with hearing loss during the COVID-19 pandemic

**DOI:** 10.1186/s12888-021-03186-2

**Published:** 2021-04-09

**Authors:** Saeed Ariapooran, Mehdi Khezeli

**Affiliations:** 1 Department of Psychology, Malayer University, Malayer, Iran; 2 Social Development and Health Promotion Research Center, Health Institute, Kermanshah University of Medical Sciences, Kermanshah, Iran

**Correction to: BMC Psychiatry 21, 114 (2021)**

**https://doi.org/10.1186/s12888-021-03118-0**

Following the publication of the original article [1], minor errors were identified in the sections. The changes have been highlighted in **bold typeface**.

Abstract:

*Results*

Among the subscales, only the Social Anxiety Disorder (39.1% in deaf vs. **9.1%** in HH, *p* = 0.009) and the School Avoidance (52.2% in deaf vs. 24.2% in HH, *p* = 0.031) significantly differed.

Background:

According to the World Health Organization, Iran is the leading country in the number of cases and deaths due to **COVID-19** in the Eastern Mediterranean region [2].

Results:

*Symptoms of ADs*

Among the subscales only SoAD (39.1% in deaf vs. **9.1% **in HH, *P*-value = 0.009) and SA (52.2% in deaf vs. 24.2% in HH, *p*-value = 0.03) significantly were higher in the deaf adolescent than HH peers.

The author group has been updated above and the original article [1] has been corrected.
